# Tobacco dependence support during and following an acute mental health inpatient admission: A cross-sectional survey of care provision in England

**DOI:** 10.18332/tpc/214129

**Published:** 2026-01-22

**Authors:** Jennifer Sweetman, Lisa Huddlestone, Heidi Stevens, Petal Petersen Williams, Emily Shoesmith, Moira Leahy, Claire Paul, Elena Ratschen

**Affiliations:** 1 Department of Health Sciences, University of York, York, United Kingdom; 2 Mental Health, Alcohol, Substance Use and Tobacco Research Unit, South African Medical Research Council, Cape Town, South Africa; 3 Institute for Life Course Health Research, Department of Global Health, Stellenbosch University, Cape Town, South Africa; 4 Sheffield Health Partnership University NHS Foundation Trust, Sheffield, United Kingdom; 5 Leeds and York Partnership NHS Foundation Trust, Leeds, United Kingdom

**Keywords:** smoking cessation, behavioral support, nicotine replacement therapy, e-cigarettes, acute mental health inpatient

## Abstract

**INTRODUCTION:**

Although significant efforts have been made to promote smoking cessation, people with mental health conditions continue to be more likely to smoke and to consume more tobacco than those without mental illness. This survey aimed to describe the provision of tobacco dependence support offered during admission and following discharge from NHS acute adult mental health wards across England.

**METHODS:**

A cross-sectional online descriptive questionnaire survey was conducted between August and September 2024 in National Health Service (NHS) Mental Health Trusts in England. Questions included identifying the Trust and role of the person responding, and characteristics of tobacco dependence support offered to patients when admitted to acute mental health wards and at discharge.

**RESULTS:**

Of the 50 Trusts approached, a response rate of 44% (n=22) was achieved. Almost all of the respondent Trusts reported offering behavioral support (n=22; 100%), nicotine replacement therapy (n=21; 95.45%), and e-cigarettes (n=21; 95.45%) during participants’ time in hospital. Tobacco dependence support offered post-discharge was reported to be in place less frequently (n=13; 59.09%); variations in the duration of support were reported. Tobacco dependence support was delivered by healthcare professionals who had completed National Centre for Smoking Cessation and Training (NCSCT) Practitioner training in all Trusts, with most Trusts also offering the NCSCT specialist Mental Health module training and/or in-house training.

**CONCLUSIONS:**

Inpatient tobacco dependence support within NHS mental health settings in England is relatively consistent; however, post-discharge care varies, presenting an opportunity to enhance smoking cessation outcomes.

## INTRODUCTION

Recently, the smoking cessation landscape in the United Kingdom (UK) has changed, driven by successive Governments’ commitment to achieving a smokefree status (prevalence <5%) within the UK by 2030^1^. A major challenge is the persistently higher smoking prevalence rates amongst people with mental health conditions^2^. This group is more likely to smoke and tend to consume more tobacco than those without mental illness^2^, increasing their risk of smoking-related health conditions and lowering life expectancy by up to 20 years^3^. To address these disparities, ongoing efforts through research, policy change, clinical guidance, and organizational collaborations are increasingly focused on improving access to tobacco dependence support and creating opportunities for individuals to change their smoking behaviors within inpatient mental healthcare settings^4-9^.

This report describes a survey of tobacco dependence support offered within NHS acute mental healthcare services in England during 2024, conducted within the context of a National Institute for Health and Care Research (NIHR) funded research program (SCEPTRE) which aims to find ways of supporting people with severe mental illness to remain smokefree after discharge from a mental health in-patient stay^10^. This survey complements the recently published Action on Smoking and Health (ASH) survey^11^, providing an opportunity to reflect on efforts to reduce the prevalence of smoking in those with mental illness. The aim was to describe the provision of tobacco dependence support offered during admission to an acute adult mental health ward and following discharge, from all NHS mental health Trusts across England.

## METHODS

### Design and participants

An online, cross-sectional survey conducted between August and September 2024, hosted on the Qualtrics platform, was sent out to tobacco leads from 50 NHS Mental Health Trusts in England. Email invitations contained the link to access the survey questions. Trusts that did not respond to invitation emails were prompted up to three times. As this survey was not classified as research, ethical review was not required.

### Measures

A questionnaire was developed by academics with input from clinicians who have experience in delivering tobacco dependence support. The survey consisted of 13 questions relating to the usual provision of tobacco dependence support offered during admission to and following discharge from a mental health inpatient stay; a copy of the survey is provided in the Supplementary file. To aid completion, questions were separated into two sections covering tobacco dependence service provision: 1) during acute adult mental health inpatient admissions, and 2) following discharge.

### Data analysis

Numerical data were summarized using descriptive statistics, and text responses were summarized thematically. Data were analyzed using SPSS version 29.0.1.0^12^ and Microsoft Excel, and were synthesized to provide a broad understanding of usual care.

## RESULTS

Twenty-two Trusts responded, achieving a response rate of 44.00%. Three Trusts provided more than one response; duplicate responses for single Trusts were combined by amalgamation. The responses from 19 (86.36%) Trusts were completed by individuals at the management level in positions such as Tobacco Dependence Service Lead or Smokefree Lead. Responses from the other three Trusts came from a Physical Health Nurse, Tobacco Dependence Advisor and Clinical Nurse treating tobacco dependence.

### Support during a mental health inpatient admission

All 22 responding Trusts (100%) reported offering Tobacco Dependence Treatment (TDT) to patients admitted for mental health inpatient care. Most Trusts reported providing TDT under a generic name such as ‘Tobacco Dependence Team’ or ‘Smokefree Team’; a small number of Trusts (n=3; 13.64%) reported providing care in line with the ‘QUIT’ program^13^ or ‘Swap and Stop’ initiative (national initiatives aimed at improving provision of TDT for people with mental health conditions).

When asked about key features of inpatient TDT provision, all responses indicated that Trusts provided behavioral support either as brief advice only (n=1; 4.55%), behavioral support delivered by a specialist advisor only (n=1; 4.55%), or both (n=20; 90.91%). In one Trust, behavioral support was delivered by ‘champions’. In addition, 21 Trusts (95.45%) indicated that nicotine replacement therapy (NRT) was offered, four Trusts (18.18%) reportedly provided varenicline, and four (18.18%) reportedly provided bupropion. E-cigarettes were reportedly provided by 21 Trusts (95.45%) with variation in whether these were provided free of charge (n=8; 36.36%), were available for sale on site (n=5; 22.73%), or a combination of those (i.e. first free and then available to purchase; n=8; 36.36%). Other support reported included: access to a smartphone app (NHS Smokefree app; n=2; 9.09%), recommending a smartphone app (n=10; 45.45%), and peer support (n=10; 45.45%). Nearly all Trusts reported offering a referral to a local Tobacco Dependence Service (TDS) on discharge from the inpatient setting (n=21; 95.45%).

Half of the NHS Trusts reported that multiple roles contributed to the provision of inpatient TDT. Specialist smoking cessation advisors contributed in 20 Trusts (90.91%); mental health workers trained in smoking cessation contributed in nine Trusts (40.91%); Pharmacists in four Trusts (18.18%), Healthy Living Advisors in two Trusts (9.09%) and other professionals including Physical Health Nurses, Champions and Ward Activity Coordinators contributed in four Trusts (18.18%).

Those individuals delivering TDT in all responding Trusts had reportedly completed the National Centre for Smoking Cessation Training (NCSCT)^14^ Practitioner training, with 21 Trusts also providing the NCSCT specialist Mental Health module training. Additionally, 16 Trusts (72.73%) offered other training, such as training in motivational interviewing and/or behavior change, in-house or local smoking cessation training.

### Support following discharge from a mental health inpatient admission

Thirteen Trusts (59.09%) reported offering post-discharge support; five of these (38.46%) reported offering ongoing support, and six of these Trusts (46.15%) offered support over a fixed period ranging between 4 weeks and 12 months. Two of the thirteen Trusts (15.38%) offered post-discharge support as part of the QUIT program, and one (7.69%) offered Swap and Stop.

Key features of post-discharge support reportedly included behavioral support provided either in person (n=8; 61.54%), over the telephone (n=11; 84.62%), via text messaging (n=4; 30.77%), other mode of delivery (n=5; 38.46%), or a combination of these 11 Trusts (84.62%). In addition, 12 Trusts (92.31%) indicated that NRT was included in the service offering, with three Trusts (23.08%) providing varenicline and two (15.38%) providing bupropion. E-cigarettes were reportedly provided free of charge by 11 NHS Trusts (84.62%). Other support reportedly included: recommending a smartphone app (n=4; 30.77%), referral to local TDS (n=9, 69.23%), and other support, including telephone follow-ups, continuity of support from inpatient advisor, and supporting the provision of NRT from other providers (n=4; 30.77%).

Four of the Trusts indicated that individuals in multiple roles contribute to this support (30.77%). Specialist smoking cessation advisors contributed in all 13 Trusts (100%); mental health workers trained in smoking cessation contributed in four Trusts (30.77%); Pharmacists in one Trust (7.69%), and other professionals, including Physical Health Nurses, Mental Health Nurse Prescribers, and support workers, provided support in two Trusts (15.38%).

The individuals delivering post-discharge support in all NHS Trusts had reportedly completed the National Centre for Smoking Cessation Training (NCSCT) Practitioner training, with 12 Trusts also providing the NCSCT specialist Mental Health module training (92.31%). Additionally, 8 Trusts (61.54%) offered other training, such as training in motivational interviewing and in-house training.

When asked whether Trust TDT service provision for people admitted to acute mental health inpatient settings met the needs of the patients, respondents from 17 of the 22 NHS Trusts (77.27%) felt that their services met the needs of their patients. Where provision was not felt to meet these needs, respondents suggested this related to funding and staffing limitations, a lack of flexibility for patients to choose support options relevant to them, and staff attitudes about smoking being a lower priority than other issues for this patient group.

## DISCUSSION

The findings of this survey align with the recent report from ASH^11^, and demonstrate that NHS Trusts are relatively consistent in offering behavioral support, nicotine replacement therapy and e-cigarettes during a mental health inpatient admission. Additionally, some Trusts incorporated or recommended smartphone apps and/or peer support during admission. Fewer NHS Trusts provided tobacco dependence support at the point of discharge from inpatient mental health care. When offered, the core components of discharge care remained consistent with those available during admission. Tobacco dependence support was provided to patients by mental health professionals who had completed NCSCT Practitioner training. Most Trusts also reported NCSCT or in-house training to enhance the knowledge and skills of professionals delivering support for tobacco dependence to individuals with mental illness. Although similar in their aims, the current survey complements the ASH survey^11^ by providing a more comprehensive overview of the types of behavior and other support offered by participating Trusts, and the training of those across a range of roles who contribute to the delivery of Tobacco dependence support.

Since guidance recommending the implementation of smokefree inpatient environments for mental healthcare in 2013^7,8^, NHS Trusts have actioned recommendations to offer behavioral support and nicotine replacement options to encourage patients to become smokefree during an inpatient admission. Efforts towards maintaining this following discharge are being implemented in some NHS Trusts; however, there remains a lack of consistency in whether support is offered, the nature and length of tobacco dependence support offered to discharged patients. With consistent post-discharge support related to more successful smoking cessation outcomes for individuals admitted to hospital settings^15^, there is potential to further improve current practice.

### Limitations

This survey is limited by the small sample size achieved; less than half of the NHS mental health Trusts in England provided a response. Self-selection of responding Trusts and reporting bias may also influence the interpretation of results and conclusions drawn.

## CONCLUSIONS

Support for tobacco dependence within NHS inpatient acute mental health settings in England is relatively consistent; however, post-discharge support varies, representing an opportunity to enhance smoking cessation outcomes.

## Supplementary Material



## Data Availability

The data supporting this research are available from the corresponding author on reasonable request.
