# Supplementary material for: Tobacco dependence support during and following an acute mental health inpatient admission: A cross-sectional survey of care provision in England
Source: Tob Prev Cessat. 2026 Jan 22;12:10.18332/tpc/214129. doi: 10.18332/tpc/214129 (PMC12825292; doi:10.18332/tpc/214129)
Supplement: Supplementary file 1 [file TPC-12-02-s1.pdf]

SUPPLEMENTARY MATERIALS

Survey questions

1. Trust name *(free-text response)*
2. Date of completion *(date)*
3. What is your job role? *(free-text response)*
4. Does your Trust offer tobacco dependence treatment to patients **admitted to acute adult mental health wards?** *(Yes/ No/ Unsure)*

   4a. If 4 is Yes, Is the tobacco dependence treatment provided by the Trust known by a particular name? For example, QUIT, Swap and Stop etc. *(Yes, please specify: free-text response/ No/ Unsure)*
5. If 4 is Yes, What are the key features of the support offered to patients **admitted to acute adult mental health wards?** Please select all that apply.

   *(**Behavioural support**: Brief advice/ Bevahioural support delivered by a specialist advisor/ Other, please specify: free-text response/ Unsure)*

   *(**Pharmacological therapy**: Licensed nicotine replacement therapy/ Varenicline/ Bupropion/ Unsure)*

   *(**E-cigarettes**: E-cigarettes provided free of charge/ E-cigarettes for sale/ Other, please specify: free-text response/ Unsure)*

   *(**Other support**: A smartphone application to support smoking cessation, Please state which smartphone application(s) are offered to support smoking cessation: free-text response/ Recommendation of an app to support smoking cessation, Please state which smartphone application(s) are recommended to support smoking cessation: free-text response/ Peer support/ Referral to local tobacco dependency service on discharge/ Other, please specify: free-text response/ None of the above/ Unsure)*
6. Who supports patients in this service? Please select all that apply. *(Specialist smoking cessation advisors/ Healthy living advisors/ Mental health worker trained in smoking cessation/ Pharmacist/ Other, please specify: free-text response/ Unsure)*
7. To what level are the individuals providing support trained? Please select all that apply. *(National Centre for Smoking Cessation Training (NCSCT) Practitioner training/ National Centre for Smoking Cessation Training (NCSCT) specialist module in mental health and smoking/ Other including any in-house training modules or sessions, please give details: free-text response/ Unsure)*
8. Does your Trust offer tobacco dependence support to adults **following discharge** from an acute mental health inpatient stay? *(Yes/ No/ Unsure)*

7a. If 7 is Yes, Is the Is the tobacco dependence treatment provided by the Trust known by a particular name? For example, QUIT, Swap and Stop etc. *(Yes, please specify: free-text response/ No/ Unsure)*

7b. How long is the post-discharge support offered for? Please select only one answer. *(Ongoing support/ Over a fixed period. Please provide the number of weeks support is provided: free-text response/ Unsure)*

9. What are the key features of the support offered to adults **following discharge** from an acute mental health inpatient stay? Please select all that apply.
*(**Behavioural support**: Behavioural support in person/ Behavioural support by phone/ Behavioural support by text-message/ Other, please specify: free-text response/ Unsure)*
*(**Pharmacological therapy**: Licensed nicotine replacement therapy/ Varenicline/ Bupropion/ Unsure)*
*(**E-cigarettes**: Free of charge e-cigarettes/ Unsure)*
*(**Other support**: A smartphone application to support smoking cessation, Please state which smartphone application(s) are offered to support smoking cessation following discharge: free-text response/ Recommendation of an app to support smoking cessation, Please state which smartphone application(s) are recommended to support smoking cessation following discharge: free-text response/ Referral to local tobacco dependency service / Other, please give details: free-text response/ None of the above/ Unsure)*

10. Who provides support to patients? Please select all that apply. *(Specialist smoking cessation advisors/ Healthy living advisors/ Mental health worker trained in smoking cessation/ Pharmacist/ Other, please give details: free-text response/ Unsure)*

11. To what level are the individuals providing support trained? Please select all that apply. *(National Centre for Smoking Cessation Training (NCSCT) Practitioner training/ National Centre for Smoking Cessation Training (NCSCT) specialist module in mental health and smoking/ Other, please give details: free-text response/ Unsure)*

12. Do you feel that the tobacco dependence treatment provision in the Trust meets the needs of service users? *(Yes, please give details: free-text response/ No, please give details: free-text response/ Unsure)*

13. Is there anything else you would like to add? *(Free-text response)*

© 2026 Sweetman J. et al.
